# Antioxidant and protective effects of extra virgin olive oil incorporated with diallyl sulfide against CCl_4_‐induced acute liver injury in mice

**DOI:** 10.1002/fsn3.2638

**Published:** 2021-10-25

**Authors:** Emna Habibi, Tarek Baâti, Leila Njim, Yassine M’Rabet, Karim Hosni

**Affiliations:** ^1^ Laboratoire des Substances Naturelles Institut National de Recherche et d’Analyse Physico‐chimique (INRAP) Sidi thabet Ariana Tunisia; ^2^ Faculté des Sciences de Gabes Université de Gabes Tunis Tunisia; ^3^ Service d’Anatomie et de Cytologie Pathologique CHU Fattouma Bourguiba Monastir Tunisia

**Keywords:** carbon tetrachloride, cellular antioxidant activity, diallyl sulfide‐rich extra virgin olive oil, functional foods, inflammation, liver injury

## Abstract

The present study delineates the effects of incorporation of 1% diallyl sulfide (DAS) into extra virgin olive oil (EVOO) on the physico‐chemical characteristics, in vitro antioxidant, and in vivo hepatoprotective properties in CCl_4_‐induced acute liver injury in mice. Results showed that the DAS‐rich EVOO exhibited good oxidative stability over one‐month storage and preserved its original quality‐related parameters including major components (oleic acid, linoleic acid, and palmitic acid), and minor components (tocopherols, chlorophylls and carotenoids, tyrosol, hydroxytyrosol, elenolic acid, oleuropein and its aglycone, pinoresinol, vanilic acid, cinnamic acid, ferulic acid, luteolin, apigenin, and sterols). Compared with EVOO or DAS, the DAS‐rich EVOO displayed the highest DPPH and ABTS‐radical scavenging activities and showed the strongest cellular antioxidant activity (CAA). In connection with its free radical scavenging activity and CAA, DAS‐rich EVOO significantly normalized the serum ALT and AST levels and prevented the increase in interleukin‐6 in CCl_4_‐intoxicated mice. The manifest anti‐inflammatory and hepatoprotective effects of DAS‐rich EVOO were further supported by liver histopathological examinations. Overall, the EVOO enrichment with DAS could open up opportunities for the development of novel functional food with improved antioxidant and hepatoprotective properties.

## Graphical abstract

The enrichment of extra virgin olive oil (EVOO) with diallyl sulfide (DAS) improves its anti‐radical, ferric reducing power, and cellular antioxidant activities. It also underpins its anti‐inflammatory and protective effect on CCl4‐intoxicated mice. The EVOO enrichment with DAS could open up opportunities for the development of novel functional food.

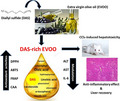

## 1 INTRODUCTION

Extra virgin olive oil (EVOO) is the key functional food of the well‐known exquisite Mediterranean diets, which are associated with lower incidences of cardiovascular diseases cancers, and immune disorders (Gambino et al., 2018). The healthy attributes of EVOO were, at least in part, attributed to its peculiar hydrophilic antioxidant compounds comprising phenyl alcohols (tyrosol and hydroxytyrosol), secoiridoids (oleuropein, oleuropein aglycone, demethyloleuropein, ligstroside, ligtroside aglycone, and elenolic acid), phenolic acids (caffeic, vanillic, *p*‐coumaric, *o*‐coumaric, protocatechuic, sinapic, *p*‐hydroxybenzoic, ferulic, and cinnamic), hydroxyl‐isochromans (1‐phenyl‐6,7‐dihydroxyisochroman and 1‐(3′‐methoxy‐4′‐hydroxy)‐phenyl‐6,7‐ dihydroxyisochroman), flavonoids (luteoline and apigenine), and lignans ((+)‐pinoresinol and (+)‐1‐acetoxypinoresinol) (El‐Riachy et al., 2011). In addition to their health beneficial effects, the EVOO antioxidants are key factors in determining the nutraceutical interest and organoleptic properties, and extending the oil shelf‐life (Reboredo‐Rodríguez et al., 2017). In particular, it was demonstrated that EVOO‐derived phenolics such as hydroxytyrosol, oleuropein aglycone, and its deacetoxy isoform prevent lipid peroxidation and improve the oxidative stability of EVOO (Carrasco‐Pancorbo et al., 2005). The ability of EVOO‐derived phenolic compounds to modulate inflammatory responses and their corresponding diseases and metabolic disorders has also been reported (Yarla et al., 2018).

However, the profitability of EVOO‐derived phenolics remains a challenging task because of their low daily intake (Rubió et al., 2012), and their quantitative and qualitative variations which in turn depend on genetic (cultivar), physiological (ripening stage at harvest), environmental (climate and soil), agronomic, and technological factors (Valls et al., 2015). Thus, to ensure an optimal intake of EVOO‐derived phenolics without affecting the energy content, the enrichment of EVOO with their own phenolics could represent an attractive option. In this context, earlier attempts showed that the enrichment of virgin olive oil (VOO) through its malaxation with extracts from olive leaves (Sánchez de Medina et al., 2011), olive pomace (Zribi et al., 2013), or a mixture of them (leaves and pomace) (Delgado‐Adámez et al., 2014) greatly enhanced its oxidative stability and antioxidant activities. Nonetheless, the main drawbacks of such enrichment strategy include increased bitterness and pungency taste, reducing consequently its sensorial properties and the consumer acceptability. Likewise, because of the pro‐oxidant properties of some phenolic compounds (e.g., pinoresinol, tyrosol, ligstroside aglycon, (+)‐1‐acetoxypinoresinol, and elenolic acid) (Carrasco‐Pancorbo et al., 2005), their incorporation into VOO could promote lipid peroxidation, which negatively influence its nutritional value and biological activities (Rubió et al., 2012). To overcome these problems, attempts have been made to produce enriched olive oils with vegetables, aromatic plants, and spices (Reboredo‐Rodríguez et al., 2017). Although the promising results in term of oxidative stability and antioxidant activities of the enriched VOO, their preparation that involves infusion, milling, and/or malaxation was usually associated with the coextraction of undesirable components such as waxes and bitters that could modify their sensory characteristics (Moldão‐Martins et al., 2004).

To conquer these drawbacks, academia and food manufacturers have employed essential oils or vegetable extracts to enrich VOO (Baiano et al., 2010). Previous enrichment assays using oregano (Asensio et al., 2013) and thyme (Miguel et al., 2014) essential oils increased the oxidative stability and consumers’ acceptability of enriched oils.

Despite these advances, the use of pure bioactive compounds for the enrichment of EVOO/VOO has not received much interest. In this frame, diallyl sulfide (DAS) (syn: allyl sulfide; diallyl thioether; oil garlic; thioallyl ether; 2‐propenyl sulfide; prop‐1‐ene‐3,3′‐thiobis; and diallylsulfane; 3‐(allylsulfanyl)‐1‐propene)), one of the most abundant garlic‐derived organosulfure compounds, which has been proved to exert protective roles in humans is considered highly promising candidate for this purpose.

The DAS is a lipid‐soluble compound responsible in part for the strong taste and odor of garlic and *Allium* plant species. It is alleged to have antioxidant (Kalayarasan et al., 2009), antimicrobial (Velliagounder et al., 2012), anticancer (Sriram et al., 2008), hepatoprotective (Shaik et al., 2008), neuroprotective (Lin et al., 2012), and anti‐inflammatory (Kalayarasan et al., 2009) activities. Owing to these biological activities, it was hypothesized that the incorporation of DAS into EVOO could greatly improve the antioxidant and hepatoprotective properties of the enriched EVOO.

Therefore, the present study was designed to produce and characterize an EVOO enriched with DAS through direct incorporation of DAS into EVOO. The DAS‐rich EVOO was futher evaluated for its in vitro antioxidant activity and its protective effect against CCl_4_‐induced hepatotoxicity in mice.

## 2 MATERIALS AND METHODS

### 2.1 Chemicals and reagents

Diallyl sulfide (DAS), carbon tetrachloride (CCl_4_), gallic acid, ferric chloride, Folin‐Ciocalteu reagent, 2,2‐azino‐bis‐(3‐ethylbenzothiozoline‐6‐sulfonic acid di‐ammonium salt) (ABTS), 2,2‐diphenyl‐1‐picrylhydrazyl (DPPH), 2,4,6‐tripyridyl‐s‐triazine (TPTZ), 6‐hydroxy‐2,5,7,8‐tetramethylchroman‐2‐carboxylic acid (Trolox), 2′,7′‐dichlorofluorescin diacetate (DCFH‐DA), 2,2′‐azobis(2‐amidinopropane)(ABAP), α‐, β‐,γ‐, and δ‐tocopherol standards were purchased from Sigma‐Aldrich (Steinheim, Germany). Physiological saline (0.9%) was procured from the Tunisian Central Pharmacy (Ben Arous, Tunisia). Phosphate buffered saline (PBS) was purchased from Gibco (Thermo Fisher Scientific Inc., Strasbourg, France), and RPMI was obtained from Gibco (BRL, Paisley, UK). Solvents of analytical and HPLC grade were purchased from Carlo Erba Reactif‐CDS (Val de Reuil, France).

### 2.2 Preparation of DAS‐rich EVOO

The monovarietal Chemlali EVOO from the crop season 2017–2018 was used. The DAS‐rich EVOO was prepared by direct addition of DAS to EVOO with the proportion of 1% (v/v). After homogenization, control and DAS‐rich EVOO were placed in closed amber glass bottles, stored at room temperature for one month, and then essayed for their physico‐chemical characteristics and biological activities.

### 2.3 Physico‐chemical analysis of DAS‐rich EVOO

The quality criteria including free acidity (% of oleic acid), peroxide value (meq O_2_/kg), conjugated dienes (K_232_), and conjugated trienes (K_270_) were determined following the official methods of analysis of the EU, EEC/2568/91 (EC Regulation 2568, 1991).

The content of chlorophylls and carotenoids in oil sample dissolved in cyclohexane was determined spectrophotometrically at 670 and 470 nm, respectively (Minguez‐Mosquera et al., 1991). Using the extinction coefficient of 613 of the major chlorophyll compound pheophytin, and 2000 as the extinction coefficient of lutein, major carotenoid of the olive oil. Pigment contents expressed as mg/kg of oil were calculated as follows:

‐ Chlorophylls (mg/kg) = (*A*_670_ × 10^6^)/(613 × 100 × *d*)
‐ Carotenoids (mg/kg) = (*A*_470_ × 10^6^)/(2000 × 100 × *d*)

Where *A*_670_ and *A*_470_ are the absorbances at 670 and 470, respectively, and *d* is the width of the cuvette (1 cm).

Fatty acid methyl esters (FAME) of the DAS‐rich oil were prepared by cold transesterification using a methanolic solution of KOH as described in the IUPAC standard procedure 2.301 (IUPAC, 1987). FAME analysis was carried out on a Hewlett‐Packard HP‐6890 gas chromatograph (Agilent Technologies, Palo Alto, CA, USA) equipped with a flame ionization detector (FID) and a polar TR‐FAMEs (Thermo Fisher Scientific Inc., Bordeaux, France) capillary column (60 m × 0.25 mm, 0.25 µm film thickness). The column temperature was initially held at 100°C for 5 min, raised to 240°C (4°C/min), and then held isotherm for 15 min. The temperature of the injector and FID detector was maintained at 240°C and 260°C, respectively. Fatty acids were identified by comparison of their retention times with those of commercial standards (Sigma‐Aldrich, Steinheim, Germany). The content of individual FAME was expressed as a percent of total peaks calculated from the electronic integration and adjusted for the internal standard.

For the determination of tocopherols, the International Standard Organisation method ISO‐9936:2016 was used. Briefly, oil (4 g) was dissolved in 25 ml hexane and filtered through a 0.45 µm PTFE filter prior to injection into a HP‐1200 HPLC system equipped with a fluorescence detector (Agilent Technologies, Rising Sun, MD, USA). Separation of tocopherols was performed using a normal phase YMC‐Pack SIL column (250 × 2 mm i.d, 5 μm particle size; YMC Co., Kyoto, Japan) with 3.85% of THF in n‐heptane at a flow rate of 1 ml/min. Tocopherols were identified and quantified at 295 and 330 nm for excitation and emission, respectively.

Regarding phenolic compounds, they were extracted and determined following the method of Fuentes et al. (2012). Briefly, sample (2.5 g) of DAS‐rich EVOO was dissolved in 5 ml hexane and extracted twice using 5 ml of 60% methanol. After vigorous agitation for 2 min, and centrifugation at 3000 × g for 10 min, hydroalcoholic phases were collected, combined, and assayed for their total phenol content and HPLC‐ESI‐MS profiling.

For the determination of total phenol content (TPC), a 200µl aliquot of sample extract or the standard caffeic acid (CA) was diluted with purified water to a total volume of 2.5 ml, mixed with 0.25 ml of 10‐fold diluted Folin‐Ciocalteu reagent and, after 3 min, with 0.5 ml of a 35% (w/v) solution of sodium carbonate, and finally diluted with purified water up to final volume of 5 ml. After 2 h incubation at room temperature and in the dark, the absorbance of the reaction mixture was measured at 725 nm (Jasco Corp., Tokyo, Japan). The TPC was expressed as mg CA equivalents per kg of oil (mg CAE/kg oil).

Profiling of phenolic compounds was performed using an Agilent 1100 series LC system (Agilent Technologies Inc., Santa Clara, CA, USA) equipped with binary pump, a photodiode array detector, a triple quadrupole MS type Micromass Autospec Ultima Pt (Waters Corp., Kelso, UK), and an ESI ion source operating in negative mode. The mobile phase consisted of A (0.1% acetic acid) and B (acetonitrile) with a flow rate of 0.25 ml/min and the injection volume was 20 µl. Separation was achieved using Superspher^®^ 100 column (12.5 cm×2 mm i.d, 4 µm, Agilent Technologies, Rising Sun, MD, USA) at 45°C using the following gradient elution program: 0.1 to 5min ‘2%B), from 5.1 to 75 min (88% B) and from 75.1 to 90 min (2% B) (Mejri et al., 2018). The UV‐Vis spectra were measured between 200 and 800 nm, and the ions in the m/z range of 100–1000 were detected using a scan time of 1 s. The operating conditions of the ESI source were as follows: capillary voltage: 3.2 KV; cone voltage: 115 V; probe temperature: 350°C; and ion source temperature: 110°C. The MS data acquisition was done with the massLynx software version 4.0 (Waters Crop., Milford, MA, USA). The tentative identification of phenolics was performed considering their UV and mass spectra, as well as by comparison of their retention time and fragmentation pattern with those of authentic standard when available and/or literature data (Ben Brahim et al., 2017; Olmo‐García et al., 2018).

### 2.4 The in vitro antioxidant activity

#### 2.4.1 DPPH radical scavenging activity

The DPPH radical activity of DAS (1% in methanol), olive oil, and DAS‐rich olive oil was determined according to the slightly modified method of Kim et al. (2005). Briefly, 100 µl of sample extract was added to 1.9 ml of 0.1 mM DPPH methanol solution. The mixture was vortexed vigorously and allowed to stand for 1h in the dark, thereafter the absorbance of the reaction mixture was measured at 515 nm and compared with the absorbance of blank control (methanol).

#### 2.4.2 ABTS‐radical scavenging activity

The ability to neutralize the radical ABTS^+^ was evaluated using the method of Yuan et al. (2005). Briefly, a 100 µl aliquot of sample extract was mixed with 1900 µl of ABTS^+^ solution, previously prepared by mixing 7 mM ABTS stock solution with 2.45 mM potassium persulfate (K_2_S_2_O_8_) and adjusted with methanol to an absorbance of 0.7 at 734 nm. After 15 min incubation, the absorbance of the reaction mixture was read at 734 nm.

#### 2.4.3 Ferric reducing antioxidant power (FRAP) assay

The procedure of Benzie and Strain (1996) was used to evaluate the ability of tested extracts to reduce Fe^3+^ to Fe^2+^. Briefly, a 100 µl aliquot of sample extract was mixed with 1900 µl of a freshly prepared FRAP solution (the working FRAP solution was prepared reacting 300 mM acetate buffer (pH 3.6), 10 mM TPTZ, and 20 mM FeCl_3_ solution (10:1:1) and preheated at 37 ˚C before use). After 30 min incubation at 37°C, the absorbance of the reaction mixture was measured at 593 nm.

In the aforementioned assays (DPPH, ABTS, and FRAP), trolox was used as a positive control and the results were expressed as µmol trolox equivalents per kg of oil (µmol TE/Kg oil).

#### 2.4.4 Cellular antioxidant activity (CAA)

For the CAA determination, the procedure described by Wolfe and Liu (2007) was used. Briefly, 100 µl of splenocytes suspension (isolated from BALB/c mice and maintained in RPMI1640 culture medium) were seeded in 96‐well microplates at a density of 5 × 10^6^/well and incubated for 24 h. After removal of the growth medium and washing of wells with 100 µl of PBS (pH 7.4), a 95 µl aliquot of extract and 5 µl of 25 mM DCFH‐DA were added and left to incubate for 1 h at 37°C. Finally, 100 µl of 600 mM ABAP in RPMI were added to all wells except for the blank, and the fluorescence was measured using a fluorescence microplate reader (Biotech, Winnoski, USA) for 1 h at 5 min interval (538 and 485 nm were used as the emission and excitation wavelength, respectively). After blank subtraction, the area under fluorescence curve versus time was integrated and the CAA value was calculated as follows:

$$\text{CAA}\left(\text{unit}\right)=\left[1‐\int \mathrm{SA}/\int \mathrm{CA}\right].$$

Where ∫SA and ∫CA are the integrated areas for sample and control curves, respectively (Wolfe & Liu, 2007).

### 2.5 The in vivo anti‐inflammatory activity

#### 2.5.1 Animals and induction of liver injury

Male Swisse albino mice (25 ± 5 g average weight) were purchased from the Tunisian Society of Pharmaceutical Industries (SIPHAT, Ben Arous, Tunisia), housed under 12 h light/dark cycle at 21 ± 2°C and 40%–60% humidity. They were fed with basal diet made up of 23% crude protein, 3% fat, 7% crude fiber, 12% moisture, 12% soybean meal, 3% corn gluten meal, 10% fish meal, 1.5% molasses, 2.5% soybean oil, 10% wheat middling, 0.5% salt, minerals, and vitamins (El Badr Co., Utique, Bizerte, Tunisia). Animals were fed ad libitum with free access to water. All animal experimentations were in accordance with guidelines by the “Comité d’Ethique de la Recherche en Sciences de la Vie et de la Santé (CER‐SVS)” at the Institut Supérieur de Biotechnologie de Monastir (ISBM), university of Monastir and with the Guide for the Care and Use of Laboratory Animals by the National Research Council. For the induction of liver injury, a single dose (5 ml/kg b.w.) of 1% CCl_4_ (dissolved in sunflower oil) was injected intraperitoneally (i.p) (Baati et al., 2012).

#### 2.5.2 Experimental design

The animals were randomized into 8 groups of 5 mice each 1) group 1 (normal control) was injected with 0.9% saline daily for 7 days; group 2 (EVOO‐treated) with EVOO daily for 7 days; group 3 (DAS‐treated) with 1%DAS in 0.9% saline daily for 7 days; group 4 (DAS‐rich EVOO‐treated) with DAS‐rich EVOO daily for 7 days; group 5 normal control injected with 1% CCl_4_ on 7th day; group 6 EVOO‐treated mice injected with 1% CCl_4_ on 7th day; group 7 DAS‐treated mice injected with 1% CCl_4_ on 7th day; and group 8 DAS‐rich EVOO‐treated mice injected with 1% CCl_4_ on 7th day.

Twenty‐four hours after CCl_4_ injection, animals were anesthetized using diethylether and sacrificed. Serum was obtained by centrifugation of the collected blood at 4000 g for 10 min and stored at 4°C for the biochemical analysis. For the histopathological examination, liver samples were dissected out and washed immediately with saline and then fixed in 10% formalin solution.

#### 2.5.3 Biochemical analyses

Serum biochemical markers such as aspartate amino transferase (AST), alanine amino transferase (ALT), creatinine, and the inflammatory cytokine interleukin‐6 (IL‐6) were determined spectrophotometrically, using commercial diagnostic kits (Biomaghreb, Ariana, Tunisia), following the manufacturer's instructions (Mejri et al., 2019).

#### 2.5.4 Liver histology

The fixed liver tissues in 10% neutral buffered formalin were gradually dehydrated in ethanol, cleared in xylene, and embedded in paraffin. Then, they were sectioned (5µm) and stained with hematoxylin‐eosin (H&E), and subsequently examined under a light microscope (Olympus, Japan).

### 2.6 Statistical analysis

Data are presented as mean ± standard error of the mean (*SEM*). Mean values for physico‐chemical parameters and antioxidant assays were compared using one‐way analysis of variance (ANOVA) followed by Tukey's post hoc test. For serum biochemical markers, the comparison between groups was performed using unpaired Student's *t* test. All analyses were performed using the Statistical Package for the Social Sciences (SPSS) software (version 18.0 for Windows, SPSS Inc., Chicago, IL, USA). Values at *p* < .05 were considered statistically significant.

## 3 RESULTS

### 3.1 Quality‐related parameters of the DAS‐rich EVOO

Table 1 summarizes the physico‐chemical properties including free acidity, peroxide value, specific UV extinction coefficients (K_232_ and K_270_), pigment content, tocopherol content, fatty acids, and phenolic composition of control and DAS‐rich EVOO. As shown, all parameters are within the range established by International Oil Council (IOC 2018) and the European community (EU Regulation 1348/2013) for EVOO. This indicates that the incorporation of DAS preserves the quality‐related parameters of the enriched EVOO. Qualitatively, the particular components of the EVOO such as the monounsaturated oleic acid, the vitamin E (α‐tocopherol), chlorophylls and carotenoids, phenyl alkohols (tyrosol, hydroxytyrosol), secoiridoids (elenolic acid, oleuropein, and its aglycone), lignan (pinoresinol), phenolic acids (vanillic acid, cinnamic acid, and ferulic acid), and flavonoids (luteoline and apigenin) have also been detected as major components of the DAS‐rich EVOO.

**TABLE 1:** Quality‐related parameters and chemical composition of EVOO‐ and DAS‐rich EVOO

	EVOO	DAS‐EVOO	E.U. 2013	IOC 2018
**Quality parameters**				
Acidity (% oleic acid)	0.62 ± 0.02^a^	0.41 ± 0.02^b^	≤0.8	≤0.8
PV (meq O2/kg)	5.39 ± 0.6^a^	4.51 ± 0.08^b^	≤20	≤20
K232 (nm)	1.20 ± 0.04^a^	1.12 ± 0.06^b^	≤2.5	≤2.5
K270 (nm)	0.15 ± 0.01^a^	0.09 ± 0.00^b^	≤0.22	≤0.22
**Fatty acids composition (%)**				
Palmitic acid C16 :0	18.62 ± 0.89^a^	15.05 ± 0.12^b^	10.887	7.5–20
Palimtoleic acid C16 :1	3,39 ± 0.08^a^	2.34 ± 0.06^b^	1.097	0.3–3.5
Stearic acid C18 :0	3,68 ± 0.04^a^	2.55 ± 0.06^b^	2.942	0.5–5
Oleic acid C18 :1	61.34 ± 2.18^a^	64.14 ± 1.65^a^	74.116	55–83
Linoleic acid C18 :2	12,35 ± 0.66^b^	14.01 ± 0.28^a^	9.955	2.5–21
Linolenic acid C18 :3	0,62 ± 0.02^b^	0.91 ± 0.03^a^	1.002	≤1
SFA	22.3 ± 1.84^a^	17.55 ± 1.44^b^	13.829	8–25
UFA	77.7 ± 2.26^b^	82.45 ± 1.26^a^	86.171	58.8–108.5
MUFA	64.73 ± 2.62^a^	66.48 ± 2.89^a^	75.213	55.3–86.5
PUFA	12,97 ± 0.72^b^	14.92 ± 1.36^a^	10.957	3.5–22
SFA/UFA	0.29 ± 0.02^a^	0.21 ± 0.01^b^	0.160	0.136–0.230
MUFA/PUFA	4.99 ± 0.02^a^	4.05 ± 0.03^b^	6.864	3.931–15.8
**Pigments (mg/kg)**				
Chlorophylls	7.32 ± 0.18^b^	11.2 ± 0.68^a^		
Carotenoids	2.26 ± 0.04^b^	5.57 ± 0.62^a^		
**Tocopherols (mg/kg)**				
α‐tocopherol	138 ± 2.6^b^	145 ± 1.4^a^		
γ‐tocopherols	5 ± 0.16^b^	7 ± 0.28^a^		
Total tocopherols	150±4^b^	161±7^a^		
**Total phenolic content (mg/kg)**	255.83 ± 12.4^b^	426,61 ± 28.44^a^		
Tyrosol (4‐HPEA) (mg/kg)	27,21 ± 1.88^b^	36.55 ± 2.62^a^		
Hydroxytyrosol (3,4‐DHPEA)	18,36 ± 0.76^b^	24.03 ± 1.46^a^		
Oleuropein (3,4‐DHPEA‐EA)	72,99 ± 4.33^b^	132.78 ± 8.13^a^		
Oleuropein aglycone	12.32 ± 0.89^b^	33.64 ± 2.64^a^		
Ligstroside (4‐HPEA‐EA)	20,58 ± 2.12^b^	67.12 ± 4.28^a^		
Oleocanthal (4‐HPEA‐EDA)	105.68 ± 6.74^b^	177.13 ± 10.61^a^		
Elenolic acid (EA)	87.22 ± 6.22^b^	101.46 ± 2.86^a^		
Pinoresinol	7.41 ± 0.69^b^	15.21 ± 1.23^a^		
Cinnamic acid	0.79 ± 0.04^b^	0.94 ± 0.02^a^		
Vanillic acid	0.35 ± 0.02^b^	0.46 ± 0.04^a^		
Ferulic acid	0.19 ± 0.01^b^	0.57 ± 0.06^a^		
Luteolin	0.51 ± 0.06^b^	0.66 ± 0.04^a^		
Apigenin	0.22 ± 0.01^b^	0.31 ± 0.02^a^		

Note

*Values are mean ± *SD* (*n* = 3); DAS, diallyl sulfide; EVOO, extra virgin olive oil; IOC, International Olive Council. Different superscript letters within a line denote significant difference at *p* < .05.

### 3.2 In vitro antioxidant activity

The antioxidant activity of control EVOO, DAS‐rich EVOO, and 1% DAS (in methanol) was evaluated through 4 complementary assays. Data from Table 2 show that incorporation of DAS into EVOO resulted in 43.3% and 209.6% increase in the DPPH and ABTS‐radical scavenging activities, respectively. In contrast, the electron‐donating ability of the DAS‐rich EVOO to reduce the ferric ion to ferrous ion was virtually lower but not significant to that of control EVOO. In all assays, the DAS was unable to scavenge the DPPH and ABTS radicals, and to reduce the ferric ions in the FRAP system. To get more realistic picture on the antioxidant activity of different samples, cellular model (CAA) that mimic the physiological conditions has been used (Table 2). As shown, DAS‐rich EVOO exhibited the highest CAA with an IC_50_ value of 116.18µg/ml, while the lowest CAA was observed for DAS (IC_50_ = 726.17µg/ml).

**TABLE 2:** In vitro antiradical, reducing power, and cellular antioxidant activities (CAA) of DAS, EVOO, and DAS‐rich EVOO

	DPPH**	ABTS	FRAP	CAA***
DAS	NA	NA	NA	726.2 ± 55.91^a^
EVOO	592.6 ± 26.81^b****^	218.5 ± 32.31^b^	1124.6 ± 93^a^	370.6 ± 61.43^b^
DAS‐rich EVOO	846.2 ± 46.33^a^	676.4 ± 27.74^a^	1064.2 ± 27^a^	116.2 ± 30.17^c^

Note

*Values are means ± *SD* (*n* = 3), DAS, diallyl sulfide; EVOO, extra virgin olive oil; NA, not active; **Results for DPPH, ABTS, and FRAP are expressed as µmol trolox equivalents per kg oil (µM TE/Kg oil); ***Results for CAA are expressed as IC_50_ (µg/ml); ****Different superscript letters within a line denote significant difference at *p* < .05.

### 3.3 Animal experiment

#### 3.3.1 Liver and renal function

**FIGURE 1:**
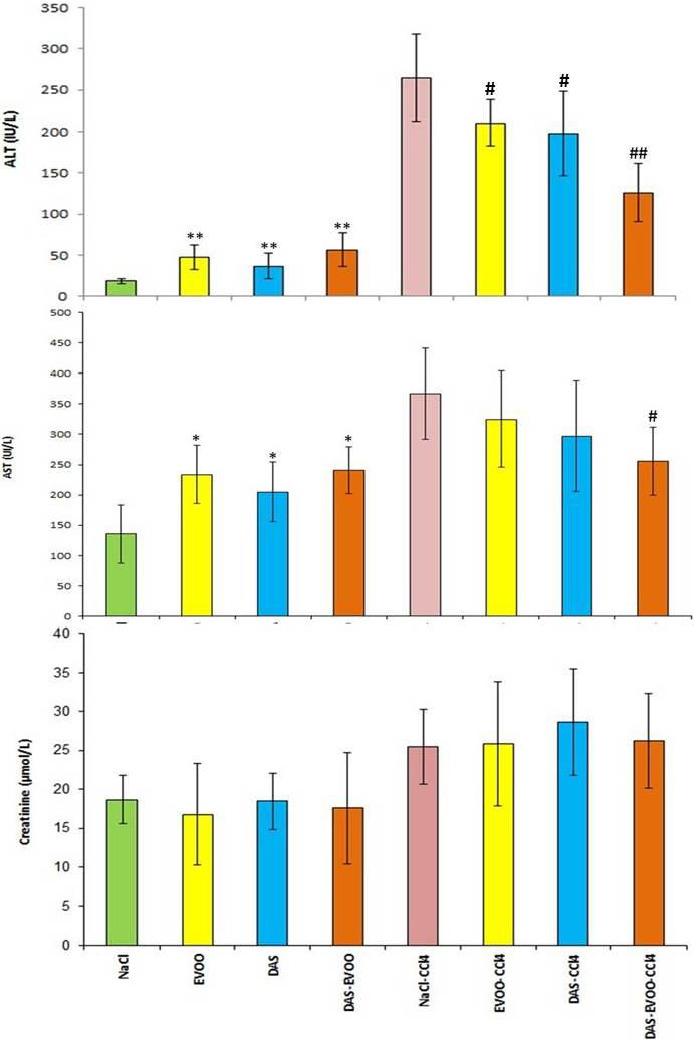
Serum ALT, AST, and creatinine levels in different experimental groups; *p < .05, **p < .01 compared with the untreated control (NaCl); ^#^ *p* < .05 and ^##^ *p* < .01 compared with the treated control (CCl_4_). DAS, diallyl sulfide; EVOO, extra virgin olive oil

Data from in vivo experiments show a significant increase in ALT and AST in CCL_4_‐intoxicated group in comparison with untreated control group (Figure 1). The i.p. injection of EVOO, 1%DAS, and DAS‐rich EVOO for 7 days before CCL_4_ injection resulted in 20.8, 25.34, and 52.42% reduction in ALT activity on CCL_4_‐induced acute liver damage, respectively. For AST activity, no obvious effects were observed in CCL_4_‐intoxicated animals treated with EVOO and 1%DAS, whereas a significant (*p* < .05) decrease in AST activity was observed for those treated with DAS‐rich EVOO. The levels of creatinine remain virtually unchanged in all animal groups indicating normal renal function.

#### 3.3.2 Pro‐inflammatory cytokine IL‐6

**FIGURE 2:**
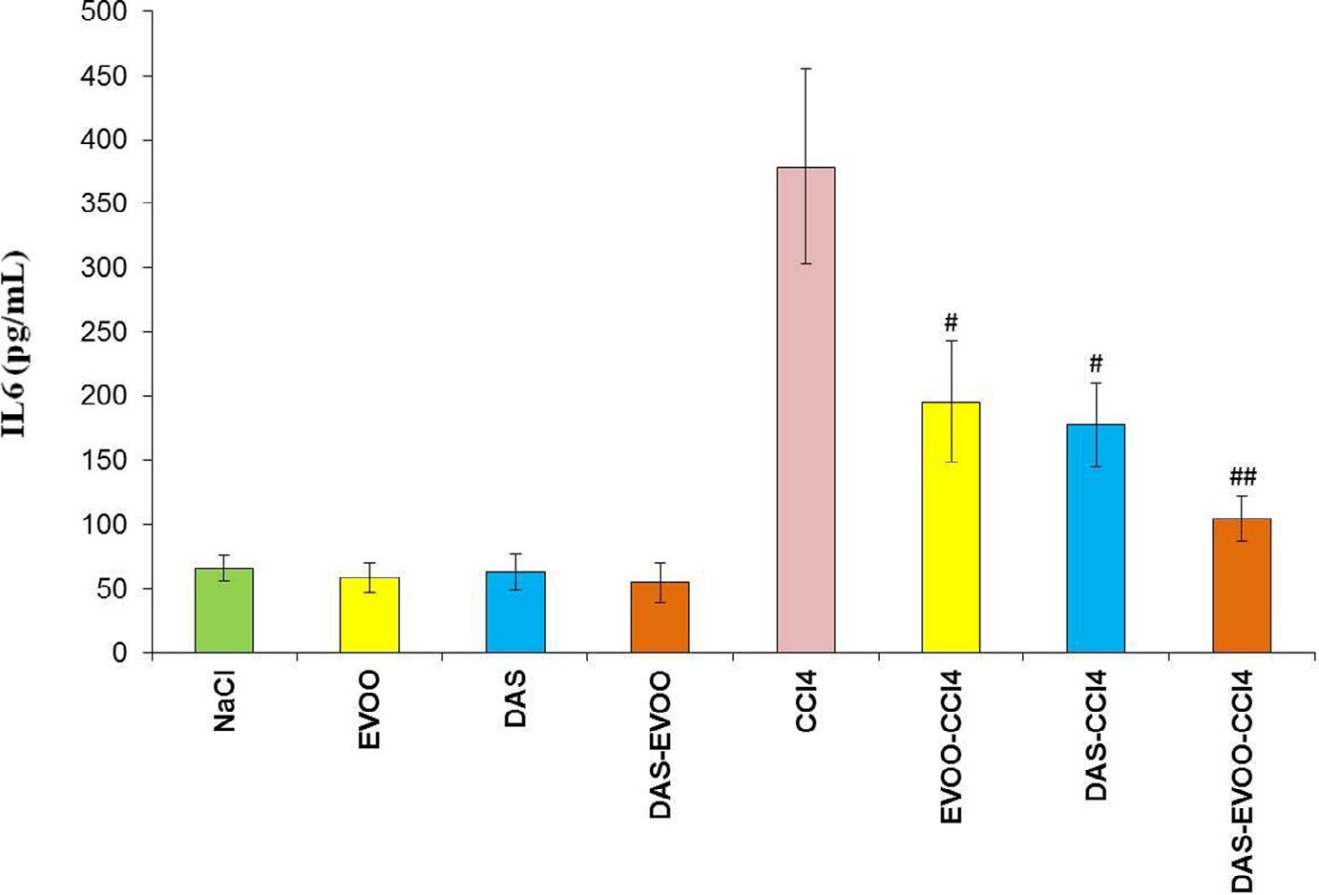
Level of IL‐6 in different experimental groups; ^#^ *p* < .05 and ^##^ *p* < .01 compared with the treated control (CCl_4_). DAS, diallyl sulfide; EVOO, extra virgin olive oil

The anti‐inflammatory effect of control EVOO, DAS, and DAS‐rich EVOO was assessed as the inhibitory capacity on CCL_4_‐induced expression of the pro‐inflammatory cytokine IL‐6 in mice. As shown in Figure 2, injection of CCL_4_ resulted in massive production of IL‐6. The CCl_4_‐induced increase in IL‐6 was significantly (*p* < .01) reduced by DAS‐rich EVOO and to a lesser extent (*p* < .05) by EVOO and DAS. Although that all treatments prevented CCl_4_‐induced IL‐6 overproduction, DAS‐rich EVOO was clearly distinguished by its high ability to inhibit IL‐6 production suggesting thereby its superior anti‐inflammatory and hepatoprotective activity.

#### 3.3.3 Liver histopathology

**FIGURE 3:**
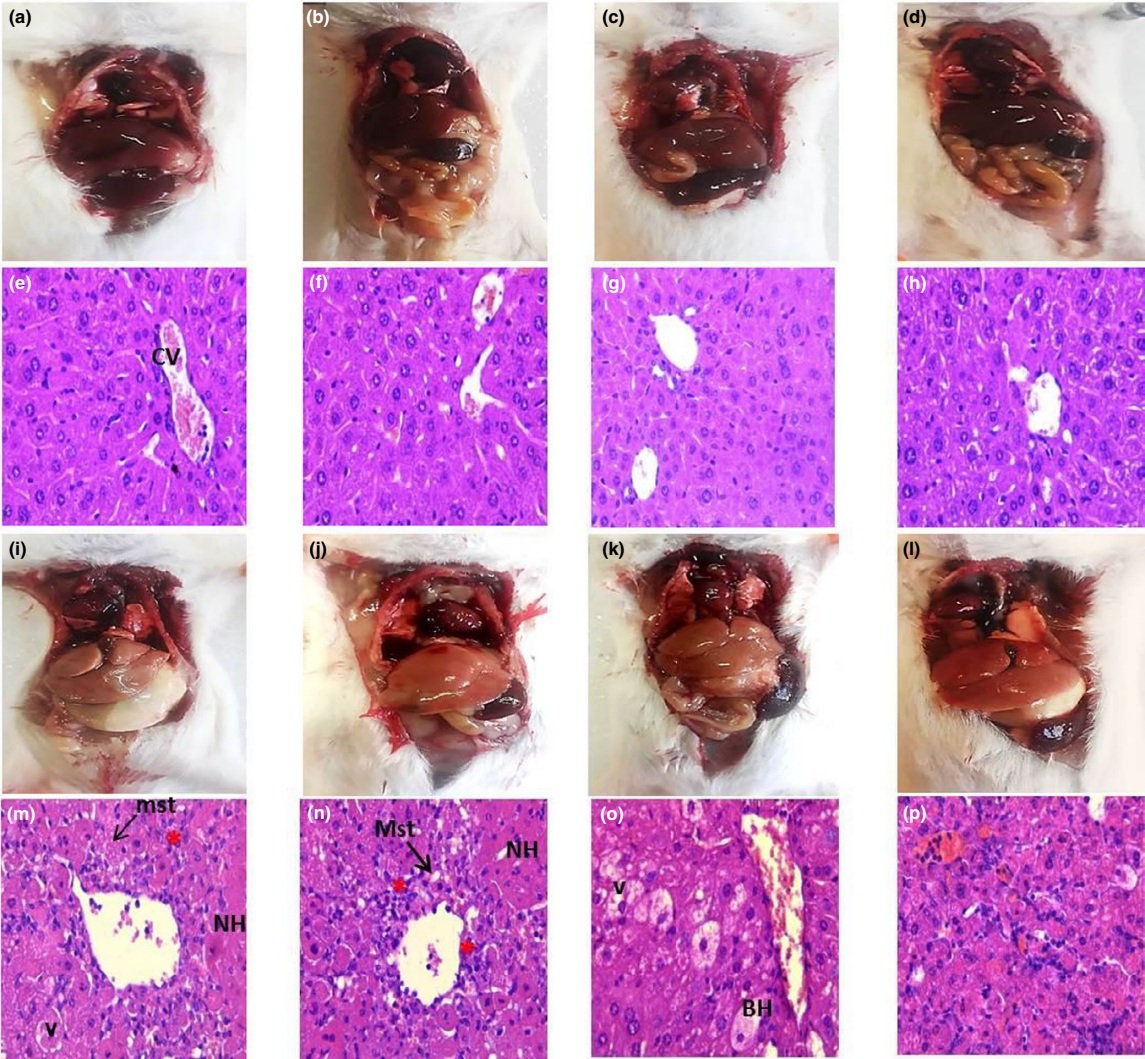
Macroscopic observation ((a–d) and (i–l)) and histopathological examination (×400 magnification) of liver from different experimental group: (a, e) Untreated control (NaCl); (b, f) Untreated control receiving EVOO; (c, g) untreated control receiving DAS; (d, h) untreated control receiving DAS‐rich EVOO; (i, m) CCl_4_‐treated control; (j, n) CCl_4_‐treated animals receiving EVOO; (k, o) CCl_4_‐treated animals receiving DAS; (l, p) CCl_4_‐treated animals receiving DAS‐rich EVOO. CV, central vein; BH, ballooned hepatocytes; NH, necrotic hepatocytes; mst, microscopic steatosis (black arrow); Mst, macroscopic steatosis (bold arrow); V, vacuolization; red asterisk, inflammatory cell infiltration

In order to confirm the hepatoprotective effect of DAS‐rich EVOO on CCl_4_‐intoxicated animals, histological assessment of liver was performed (Figure 3). Macroscopic observation revealed normal aspect of the brown liver in untreated control and untreated control receiving EVOO, DAS, or DAS‐rich EVOO (Figure 3a–d). Histologic examination of hepatic samples of these groups showed normal architecture with narrow sinusoids and well‐ordered hepatocytes with a prominent nuclei and granulated cytoplasm and surrounded by a distinct cell border (Figure 3e–h). The intraperitoneal injection of CCl_4_ caused severe injury as revealed by the visible pale liver (Figure 3i). Histopathological study of liver tissues from CCl_4_‐intoxicated group showed classic histopathological lesions characterized by severe hepatocyte degeneration and necrosis, hepatocytes vacuolization and ballooning, congestion, inflammatory cell infiltration, and macroscopic and microscopic steatosis (Figure 3 m). These morphologic and histologic changes were markedly attenuated (well‐shaped hepatocyte with well‐defined nuclei, increased intercellular space, granulated cytoplasm, and well‐defined cell wall) in CCl_4_‐intoxicated group treated with DAS‐rich EVOO (Figure 3p) suggesting its potent hepatoprotective effect. The recovery of liver architecture was less evident with EVOO and DAS treatments (Figure 3n, o).

## 4 DISCUSSION

In recent years, several landmark studies have shown that enrichment of EVOO/VOO can improve their oxidative stability, nutritional quality, and organoleptic properties (Benkhoud et al., 2021; Rubió et al., 2012). In this study, it has been demonstrated that the enrichment of EVOO with DAS preserves its quality in term of major (e.g., fatty acids) and minor compounds (e.g., phenolics, pigments, sterols, and vitamin E), probably by inhibiting lipid peroxidation and the formation of free radicals. This result indicates that the incorporation of DAS into EVOO effectively maintains its antioxidant properties as appreciable amounts of antioxidants (e.g., tocopherols, phenolics, chlorophylls, and carotenes) were detected. Supports to this assumption are given by Kim et al. (1997) who reported that the diallyl disulfide (DADS)‐ and diallyl trisulfide (DATS)‐rich garlic homogenates effectively inhibit lipid oxidation in an oil‐water system. Six years later, Yin and Cheng (2003) showed that exogenous application of DAS significantly delayed lipid oxidation in ground beef owing to its reducing or chelating ability.

To confirm the DAS‐mediated protective effect against lipid oxidation, the antioxidant activity of EVOO, DAS, and DAS‐rich EVOO samples was assessed through three complementary assays: DPPH, ABTS, and FRAP. Key results showed that DAS‐rich EVOO had superior antiradical activities in comparison with the EVOO sample which was distinguished by its greater ferric reducing ability. The DAS sample was ineffective in the three assays probably because of its low concentration, and/or low affinity toward free radicals and ferric ions. This concurs with the results of Yin et al. (2002) who showed that DAS is not effective in chelating ferrous iron and preventing lipid oxidation in the liposome system.

In the cellular model, the highest activity was again observed for the DAS‐rich EVOO while the lowest was observed for DAS. At this point, it can inferred that DAS incorporation into EVOO potentiates its antioxidant activity presumably through (i) synergistic action between DAS and antioxidants originally present in EVOO such as phenolic acids (vanillic acid, cinnamic acid, and ferulic acid), secoiridoids (elenolic acid, oleuropein, and its aglycone), lignan (pinoresinol), phenyl alkohols (tyrosol and hydroxytyrosol), vitamins (α‐tocopherol), flavonoids (luteoline and apigenine), and pigments (carotenoids and chlorophylls) and/or (ii) its protective effect against oxidation/degradation of natural EVOO antioxidant compounds. Previous studies showed that the antioxidant performance of DAS in meat and liposomes was greatly improved when combined with α‐tocopherol (Kim et al., 1997; Yin et al., 2002). The effects of DAS as synergist was further confirmed in gentamycin‐induced nephrotoxicity in Wistar rats (Kalayarasan et al., 2009). The authors attributed the synergistic antioxidant activity to the sparing effect of DAS on lipophilic antioxidants such as α‐tocopherol and β‐carotene (Liao & Yin, 2000). In the present study, the enhanced antioxidant activity of DAS‐rich EVOO is likely attributed to the combined effects of DAS and other lipophilic antioxidants present in EVOO which might be more beneficial for lipid stability. From practical standpoint, the incorporation of DAS into EVOO could effectively improve its antioxidant activity and extend its oxidative stability.

Prompted by these auspicious results, and given the intriguing biological activities (i.e., anticancer, antidiabetic, hepatoprotective, cardioprotective, neuroprotective, and lipid regulating properties, among others (Hassen et al., 2015) of some identified components in DAS‐rich EVOO, we have next assessed its protective effects against CCl_4_‐induced liver injuries in mice. Results of the bioassays showed that i.p. injection of CCl_4_ caused acute liver damage characterized by increased serum ALT and AST activities suggesting the alteration of hepatic function (Figure 1). Pale liver (Figure 3i) with necrotic, degenerated, and ballooned hepatocytes, as well as congestion, inflammatory cells infiltration and macroscospic, and microscopic steatosis (Figure 3m) were found as the main hallmarks of CCl_4_‐intoxication. These macro/microscopic alterations are often associated with increased oxidative/nitrosative burst induced by excessive production of free radicals (Domitrović et al., 2012).

In general, these symptoms are typical to CCl_4_‐induced hepatotoxicity which was mainly due to the cytochrome P450‐induced production of the harmful trichloromethyl radical (CCl_3_ **^•^)** and trichloromethyl peroxy radical (CCl_3_OO•). The trichloromethyl‐free radicals could induce lipid peroxidation and the destruction of polyunsaturated fatty acids especially those associated with phospholipids leading ultimately to the disruption of cell and mitochondrial membranes (Pan et al., 2018). As immediate consequences, an increased permeability and enhanced release of ALT and AST considered as indicators of liver cell membrane and liver mitochondrial damage, respectively. The i.p. injection of EVOO and DAS restored the levels of ALT without effect on AST activity. In contrast, DAS‐rich EVOO not only normalize the ALT levels, but also reduce those of AST suggesting that DAS‐rich EVOO had protective effects on CCl_4_‐intoxicated mice through liver cell membrane stabilization.

Microscopic examinations underpin these observations and clearly showed the recovery of liver appearance (normal brown liver) and histologic parameters including the well‐shaped hepatocytes with well‐defined cell wall, prominent nuclei, granulated cytoplasm, etc. (Figure 3p). These ameliorative effects were not recorded for EVOO and DAS treatments (Figure 3n). Given its superior antioxidant activity in vitro, it can be suggested that the hepatoprotective effects of DAS‐rich EVOO against CCl_4_‐induced liver damage are mediated through its ability to neutralize the free radicals (CCl_3_ **^•^** and CCl_3_OO•), prevent lipid peroxidation, maintain the functional integrity of liver biomembranes, and reduce enzymes leakage. The decrease in relevant pro‐inflammatory cytokine IL‐6 is suggested too. Data from Figure 2. showing that DAS‐rich EVOO was more effective in reducing IL‐6 than that of EVOO and DAS provide support for such assumption. The mechanism behind the inhibitory effect of DAS‐rich EVOO on IL‐6 production is understood, but may be likely associated with its capacity to interfere with the expression of nuclear factor kappa B (NF‐κB) which is responsible for the expression of IL‐6 (Zhou et al., 2009). Additional in depth experiments are needed to decipher the exact mechanism involved in such inhibitory effect of DAS‐rich EVOO.

The anti‐inflammatory activity of some EVOO components has been extensively investigated (Cárdeno et al., 2013; Cicerale et al., 2012). For example, the secoiridoids oleuropein and it aglycone have been found to exert their anti‐inflammatory effect *via* inhibition of lipid peroxidation, and cyclooxygenase enzymes (COX‐1 and COX‐2), as well as the reduction in production of tumor necrosis factor‐α (TNF‐α), and pro‐inflammatory cytokines (IL‐1β and IL‐6) in a mouse model of carrageenea‐induced pleurisy (Impellizzeri et al., 2011). Later, it has been demonstrated that oleuropein effectively suppressed oxidative/nitrosative liver damage by inhibiting NF‐κB, inducible nitric oxide synthase (iNOS), and 3‐nitrotyrosine (3‐NT) versus a marked activation of caspase‐3 in CCl_4_‐intoxicated mice (Domitrović et al., 2012). In the corresponding study, the hepatoprotective effect of oleuropein was found to be mediated through the nuclear factor erythroid 2‐related factor (Nrf2)‐mediated induction of heme oxygenase‐1 (HO‐1). These mechanisms in addition to the radical scavenging activity and activation of endogenous enzymatic antioxidants, as well as the reduction in the production of eicosanoids have been proved for hydroxytyrosol, tyrosol (Rosignoli et al., 2013), oleocanthal (Scotece et al., 2012), caffeic acid (Miles et al., 2005), luteolin (Yan et al., 2019), apigenin (Yue et al., 2020), vanillic acid (Itoh et al., 2010), cinnamic acid (Fernández‐Martínez et al., 2007), pinoresinol (Kim et al., 2010), oleic acid (Tanaka et al., 2009), α‐tocopherol (Yachi et al., 2010), β‐sitosterol (Devaraj et al., 2020), and β‐carotene (Clugston, 2020) in in vitro and in vivo models (Cárdeno et al., 2013). In the present study, it is possible that DAS and one or more bioactive components of the EVOO will synergistically contribute to the protective effect of DAS‐rich EVOO against CCl_4_‐induced hepatotoxicity, which is in tune with our previous conclusions regarding the in vitro antioxidant activity. Synergy involving DAS has been previously observed with pomegranate fruit extract (PFE) (George et al., 2011). In their experimental model, authors showed that DAS and PFE impart better cancer chemopreventive effect than either of these agents alone because of the enhanced antioxidant and anti‐inflammatory of the system DAS/PFE.

Particularly, striking is the low protective effect of EVOO and DAS in CCl_4_‐intoxicated mice, despite their well‐known anti‐inflammatory and hepatoprotective properties (Kalayarasan et al., 2009). These unexpected results might be due to the low concentrations used and/or metabolization of the bioactive components.

## 5 CONCLUSION

The key results of this study revealed that the incorporation of DAS preserves the original composition and enhanced the oxidative stability of the DAS‐rich EVOO. It also improves its antioxidant activity in vitro and underpins its anti‐inflammatory and hepatoprotective effect on CCl_4_‐intoxicated mice, which was shown by the decrease in serum ALT, AST, and creatinine levels, as well as improved liver histology. Further studies are needed to find out the exact mechanism of heptaoprotective action of DAS‐rich EVOO, and its exploitation for the prevention and treatment of liver injury.

## CONFLICTS OF INTEREST

The authors declare that there are no conflicts of interest.

## AUTHOR CONTRIBUTIONS

**Emna Habibi:** Data curation (equal); Investigation (equal); Methodology (equal); Writing‐original draft (supporting). **Tarek Baâti:** Conceptualization (supporting); Data curation (equal); Investigation (equal); Methodology (equal); Supervision (supporting); Visualization (equal). **Leila Njim:** Data curation (supporting); Methodology (supporting); Visualization (supporting). **Yassine M'Rabet:** Data curation (equal); Methodology (equal); Visualization (equal). **karim hosni:** Conceptualization (lead); Data curation (supporting); Investigation (supporting); Supervision (lead); Validation (supporting); Visualization (supporting); Writing‐original draft (lead); Writing‐review & editing (lead).

## ETHICS APPROVAL

This study was performed in strict accordance with protocols approved by the Ethical Committee for the Research in Life and Health Sciences (CER‐SVS) at the High Institut of Biotechnology of Monastir (ISBM) of the University of Monastir and complied with the Guide for the Care and Use of Laboratory Animals by the National Research Council (NIH, 8th Edition)..

## Data Availability

The data that support the findings of this study are available from the corresponding author upon reasonable request.
